# Cardiovascular risk of Janus kinase inhibitors compared with biologic disease-modifying antirheumatic drugs in patients with rheumatoid arthritis without underlying cardiovascular diseases: a nationwide cohort study

**DOI:** 10.3389/fphar.2023.1165711

**Published:** 2023-10-30

**Authors:** Yun-Kyoung Song, Gaeun Lee, Jinseub Hwang, Ji-Won Kim, Jin-Won Kwon

**Affiliations:** ^1^ College of Pharmacy, Daegu Catholic University, Gyeongsangbuk-do, Republic of Korea; ^2^ Department of Statistics, Daegu University, Gyeongsangbuk-do, Republic of Korea; ^3^ Division of Rheumatology, Department of Internal Medicine, Daegu Catholic University School of Medicine, Daegu, Republic of Korea; ^4^ BK21 FOUR Community-Based Intelligent Novel Drug Discovery Education Unit, College of Pharmacy and Research Institute of Pharmaceutical Sciences, Kyungpook National University, Daegu, Republic of Korea

**Keywords:** janus kinase inhibitors, biologic DMARDs, cardiovascular risk, asian, rheumatoid arthritis

## Abstract

**Objectives:** Despite the ethnic differences in cardiovascular (CV) risks and recent increase in the prescription of Janus kinase (JAK) inhibitors, limited evidence is available for their CV outcomes in Asian patients with rheumatoid arthritis (RA). We aimed to compare the major adverse CV events (MACEs) of JAK inhibitors to those of biologic disease-modifying antirheumatic drugs (bDMARDs) in Korean patients with RA without baseline CV disease (CVD).

**Methods:** In a nationwide retrospective cohort study, patients newly diagnosed with RA without a history of CVD between 2013 and 2018 were identified using the National Health Insurance Service database. The cohort was followed up until the end of 2019 for the development of MACEs. Hazard ratios (HRs) for MACEs such as myocardial infarction, stroke, coronary revascularization, or all-cause death, were estimated using Cox proportional hazard regression in a propensity score-matched cohort.

**Results:** In total, 4,230 matched patients with RA were included (846 JAK inhibitor users and 3,384 bDMARD users). The crude incidence rate (95% confidence intervals, CI) per 100 patient-years for MACEs was 0.83 (0.31–1.81) and 0.74 (0.53–1.02) in the JAK inhibitor and bDMARD groups, respectively. The risk of MACEs was not significantly different between JAK inhibitor and bDMARD users with an adjusted HR (95% CI) of 1.28 (0.53–3.11). There were no significant differences in the risk of MACEs between JAK inhibitors and bDMARDs in each subgroup according to the types of bDMARDs, age, sex, Charlson comorbidity index score, and comorbidities.

**Conclusion:** Compared to bDMARDs, JAK inhibitors were not associated with the occurrence of MACEs in Korean patients with RA without a history of CVD.

## 1 Introduction

Rheumatoid arthritis (RA) is associated with an increased risk of cardiovascular (CV)-related morbidity and mortality, possibly due to the chronic, systemic immune-mediated inflammation (Avina-Zubieta et al., 2012; Smolen et al., 2018). Disease-modifying antirheumatic drugs (DMARDs), including conventional, biologic or targeted synthetic DMARDs, are mainly used for lifetime management of RA, among which Janus kinase (JAK) inhibitors targeting JAK family kinases offer an important alternative to biologic DMARDs (bDMARDs) (Smolen et al., 2018; Takabayashi et al., 2021). The recent European League Against Rheumatism (EULAR) guideline recommends JAK inhibitors for patients with poor prognostic factors who fail to achieve the treatment target with initial treatment with conventional synthetic DMARDs (csDMARDs) along with bDMARDs (Smolen et al., 2020). Three JAK inhibitors are currently available for the clinical management of RA since the first approval of tofacitinib approximately 10 years ago, and then baricitinib and upadacitinib approximately 3–4 years ago in the United States (US) and Korea (US Food & Drug Administration; Korean Ministry of Food and Drug Safety).

However, increasing evidence suggests that JAK inhibitors are unsuitable for patients at risk for thromboembolic or CV events because they may negatively impact thrombopoietin signaling and platelet homeostasis by blocking the intracellular signaling pathways of inflammatory cytokines (Gadina et al., 2019; Baldini et al., 2021; Song et al., 2022; Ytterberg et al., 2022). Nevertheless, the association between JAK inhibitors and CV outcomes is unclear. Several studies, including randomized controlled trials (RCTs) and large population-based cohorts, have shown that JAK inhibitors do not have a significant impact on the risk of major adverse CV events (MACEs) in patients with RA regardless of their underlying CV risk (Xie et al., 2019b; Khosrow-Khavar et al., 2022; Taylor et al., 2022). However, a recent large-scale RCT reported an increased risk of MACEs with tofacitinib compared to that with a tumor necrosis factor (TNF) inhibitor in patients aged ≥50 years with RA and CV risk factors (Ytterberg et al., 2022). Therefore, the regulatory authorities recommend restricting the use of JAK inhibitors in patients with risk factors for CV disease (CVD) and those with a history of smoking (US Food and Drug Administration, 2021a; European Medicines Agency, 2022; Korean Ministry of Food and Drug Safety, 2022). However, this recommendation cannot be directly applied to patients aged <50 years and those without underlying CVD (Singh, 2022). Moreover, most studies on the impact of JAK inhibitors on MACEs have included Western populations. Despite the ethnic differences in CV risks and mortality between the Asian and Western populations and recent increase in the prevalence of RA and prescription of JAK inhibitors in Korea, limited evidence is available for the CV outcomes of JAK inhibitors in the Asian population (Won et al., 2018; Health Insurance Review and Assessment Service, 2022; Tsao et al., 2022). Very recently, a cohort study was conducted in the Asian patients with RA to assess the CV risks of JAK inhibitors and showed no difference in the risk compared to TNF inhibitors (Tong et al., 2023). Nevertheless, to the best of our knowledge, there is a lack of studies comparing CVD risk between JAK inhibitors and bDMARDs in Asian RA patients without a history of CVDs.

Successful control of RA with JAK inhibitors while minimizing its negative effects on CVD is clinically important. Therefore, this study aimed to compare the CV risk of JAK inhibitors and bDMARDs in Korean patients newly diagnosed with RA without baseline CVD.

## 2 Materials and methods

### 2.1 Study design and data source

This cohort study was performed using national insurance reimbursement claims data from 2011 to 2019, which were officially provided by the Health Insurance Review and Assessment Service (HIRA) of Korea. The HIRA is an independent and public insurance agency that reviews medical fees, evaluates whether the prescribed drugs are medically necessary on the basis of drug labels, and provides national insurance coverage to 97.1% Korean citizens (Health Insurance Review and Assessment Service, 2021a; Health Insurance Review and Assessment Service, 2021b). The data included information on demographics, diagnosis, procedure, and prescription, with an unidentifiable code representing each individual. This study was approved by the Institutional Review Board (IRB) of Daegu Catholic University (IRB No. CUIRB-2019-E012, 25 September 2019), which waived the requirement for informed consent because all patient data were anonymized and de-identified by a randomized identification number prior to retrospective analysis.

### 2.2 Study population

**FIGURE 1:**
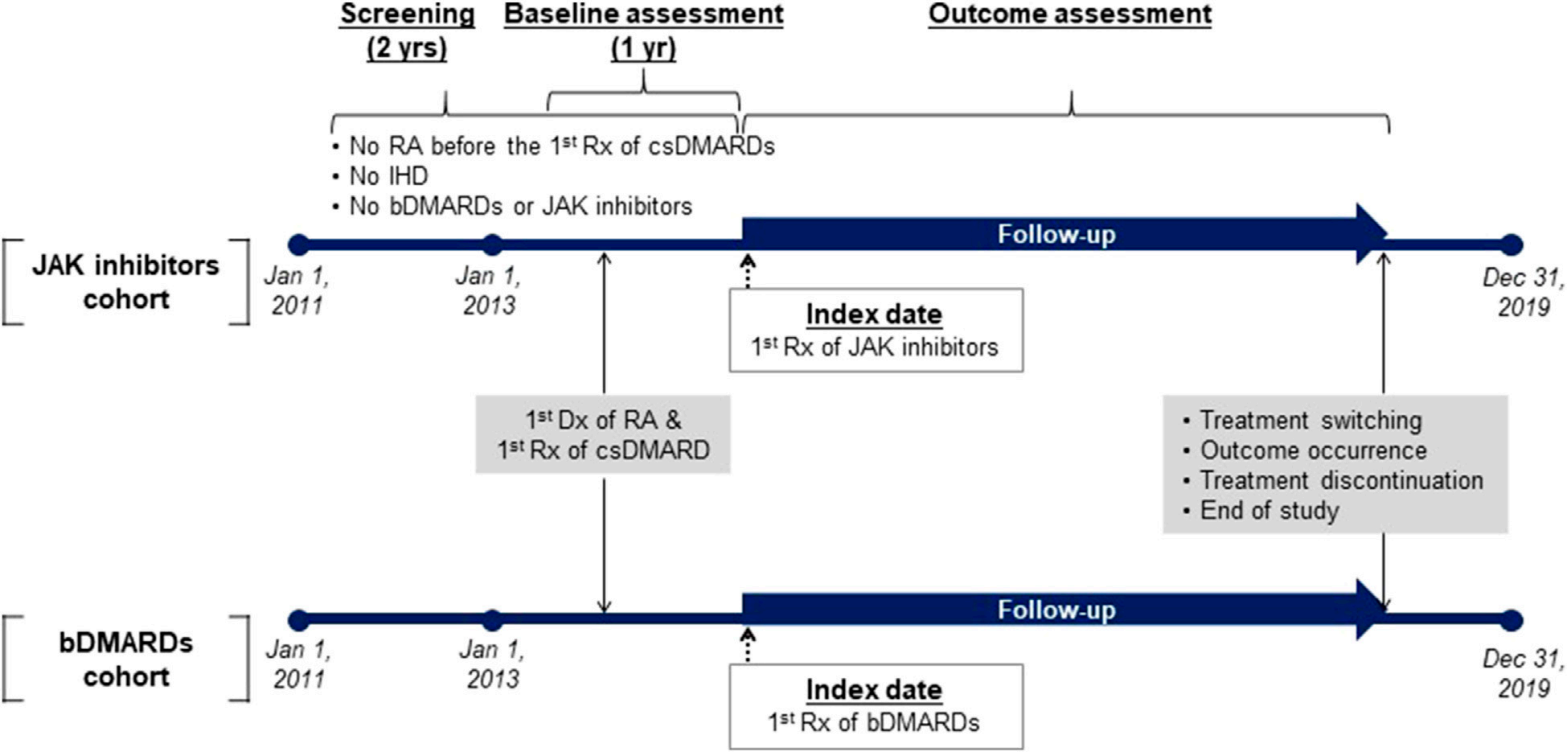
Study timeline. Abbreviations: bDMARD, biologic disease-modifying antirheumatic drug; csDMARD, conventional synthetic disease-modifying antirheumatic drug; Dx, diagnosis; IHD, ischemic heart disease; JAK, Janus kinase; RA, rheumatoid arthritis; Rx, prescription.

Adult patients who were first diagnosed with RA using the diagnostic codes of M05 or M06 in accordance with the International Classification of Diseases and Related Health Problems, 10th revision (ICD-10) between 1 January 2013 and 31 December 2018 and were prescribed at least one csDMARD (hydroxychloroquine, methotrexate, sulfasalazine or leflunomide) on the first day of RA diagnosis according to claims data were eligible for inclusion (World Health Organization, 2019). As shown in Figure 1, the index date was defined as the first prescription date of bDMARDs (including TNF inhibitors [such as infliximab, etanercept, adalimumab, golimumab, and certolizumab pegol] and non-TNF inhibitors [such as rituximab, abatacept, anakinra, and tocilizumab]) or JAK inhibitors (including tofacitinib and baricitinib). Upadacitinib, which was first used in Korea in 2020, could not be included in this study considering the study design. Users who had not received bDMARDs or JAK inhibitors within the last 2 years were defined as new users of bDMARDs or JAK inhibitors. Patients were excluded if they were (1) <20 years old on the index date; (2) diagnosed with RA 2 years before the first prescription of csDMARDs; (3) diagnosed with rheumatic heart disease (ICD-10 codes: I00−I09), ischemic heart disease (ICD-10 codes: I20−I25), valve disorders (ICD-10 codes: I34−I36), heart failure (ICD-10 codes: I50), or stroke (ICD-10 codes: I60−I69) within 2 years before the index date; (4) prescribed csDMARDs only once before the index date considering eligible patients for the use of JAK inhibitors or bDMARDs based on the EULAR guideline or Korean insurance coverage criteria; (5) diagnosed with only adult-onset Still disease (ICD-10 codes: M06.1) or inflammatory polyarthropathy (ICD-10 codes: M06.4) to include only patients with a diagnostic code for RA; (6) diagnosed with cancer (ICD-10 codes: C00−C99) during the study period which might affect the study outcomes; and (7) diagnosed with ankylosing spondylitis (ICD-10 codes: M45), Crohn’s disease (ICD-10 codes: K50), ulcerative colitis (ICD-10 codes: K51), psoriatic arthritis (ICD-10 codes: M07.0−M07.3), or psoriasis (ICD-10 codes: L40) for which JAK inhibitors or bDMARDs could be used (Kim et al., 2011; Health Insurance Review and Assessment Service, 2019; Smolen et al., 2020). For the latter two exclusions, all available data between 2011 and 2019 were used to clearly evaluate the study outcomes (Kim et al., 2011). Ultimately, our intention was to include naïve users for JAK inhibitors or bDMARDs among newly diagnosed patients with RA who had no history of CVDs. The baseline period was used for assessing comorbidities, comedications, and confirming new use of bDMARDs or JAK inhibitors.

### 2.3 Exposure data

Exposure was determined by the prescription date and number of days of drug supply. The dosing intervals for bDMARDs administered via infusion was determined based on the drug label (Korean Ministry of Food and Drug Safety, 2022). Patients were grouped into JAK inhibitor and bDMARD groups according to initial prescription and followed up thereafter. Patients were followed-up from the day after the index date and to the date of the following censoring events, whichever occurred first: 1) index drug discontinuation defined as treatment gap >365 days between its prescriptions, 2) switching to a JAK inhibitor in the bDMARD group or a bDMARD in the JAK inhibitor group, 3) outcome occurrence, and 4) end of the study (31 December 2019). The follow-up period for each patient varied depending on the patient’s entry date. Switching to a different JAK inhibitor or bDMARD was permitted in the JAK inhibitor and bDMARD groups, respectively.

### 2.4 Study outcomes

The primary outcome was a composite MACE of myocardial infarction (MI, ICD-10 codes: I21), ischemic stroke (ICD-10 code: I63), coronary revascularization such as angioplasty or bypass surgery (procedure codes: M6551–M6554, M6561–M6567, M6571, M6572, M6620, M6634, M6638, O1640–O1642, O1647–O1649, OA640–OA642, and OA647–OA649) or all-cause death (claims related to death as a medical result) (Kip et al., 2008). The secondary outcomes included each component of the MACEs. The date of the first occurrence of any of the above four components was defined as the date of composite CV outcomes. In addition, we considered hospitalization for MI, stroke, or coronary revascularization and diagnosis of stroke based on brain imaging including computed tomography (CT) or magnetic resonance imaging (MRI) to validate the clinical outcomes (Yeom et al., 2015; Park et al., 2019).

### 2.5 Confounding variables

During the 365-day baseline period prior to the index date, the following baseline characteristics, which were considered to be potentially associated with the study outcomes and RA severity, were assessed: age at the index date, sex, index year, type of insurance, Charlson comorbidity index (CCI) score, comorbidities (e.g., dyslipidemia, diabetes mellitus, hypertension, osteoporosis, anemia and eye disorders), medications for RA (e.g., csDMARDs, corticosteroids, non-steroidal anti-inflammatory drugs [NSAIDs], and tramadol), and comedications (e.g., statins, antidiabetics and antihypertensives). The adjusted model included covariates such as age, sex, index year, type of insurance, CCI score, and comorbidities (except diabetes mellitus). Age was included as the categorical variable in the final model.

### 2.6 Statistical analysis

Propensity score (PS) matching was performed to adjust for the effect of confounding variables between the JAK inhibitor and bDMARD groups. The PS was estimated using logistic regression with variables including age, sex, index year, type of insurance, medications for RA, CCI score, comorbidities, and comedications. JAK inhibitor users were matched 1:4 to bDMARD users using the greedy 5-to-1 digit matching algorithm (Parsons, 2001). Distribution of propensity score before and after matching was examined using a standardized difference, with a value exceeding 0.1 considered indicative of an imbalance.

Data are shown as numbers and percentages for categorical variables and medians and ranges for continuous data. Fisher’s exact test and the chi-square test were used to compare categorical data, while the unpaired *t*-test and Mann-Whitney U test were used to compare continuous data. Incidence rates (IRs) and 95% confidence intervals (CIs) were calculated for primary and secondary outcomes in the PS-matched study cohort. Cox proportional hazard regression was used to estimate hazard ratios (HRs) and 95% CI for study outcomes according to the use of JAK inhibitors or bDMARDs. The proportionality assumption in the Cox proportional hazard model was examined using the goodness-of-fit test.

Subgroup analysis was performed according to the type of bDMARD (TNF inhibitors only and others), age (<65 and ≥65 years), sex, CCI score, and presence of CVD-related comorbidities (such as hypertension or dyslipidemia) or RA-related comorbidities (such as eye disorders, osteoporosis, or anemia). Sensitivity analyses were performed to evaluate the robustness of the primary analysis results under the modifications of the permissible treatment gap of 90 and 180 days (US Food and Drug Administration, 2013). Statistical significance was set at a two-sided *p*-value of <0.05. All statistical analyses were performed using SAS version 9.4 (SAS Institute, Cary, NC, United States of America).

## 3 Results

### 3.1 Demographic characteristics

**FIGURE 2:**
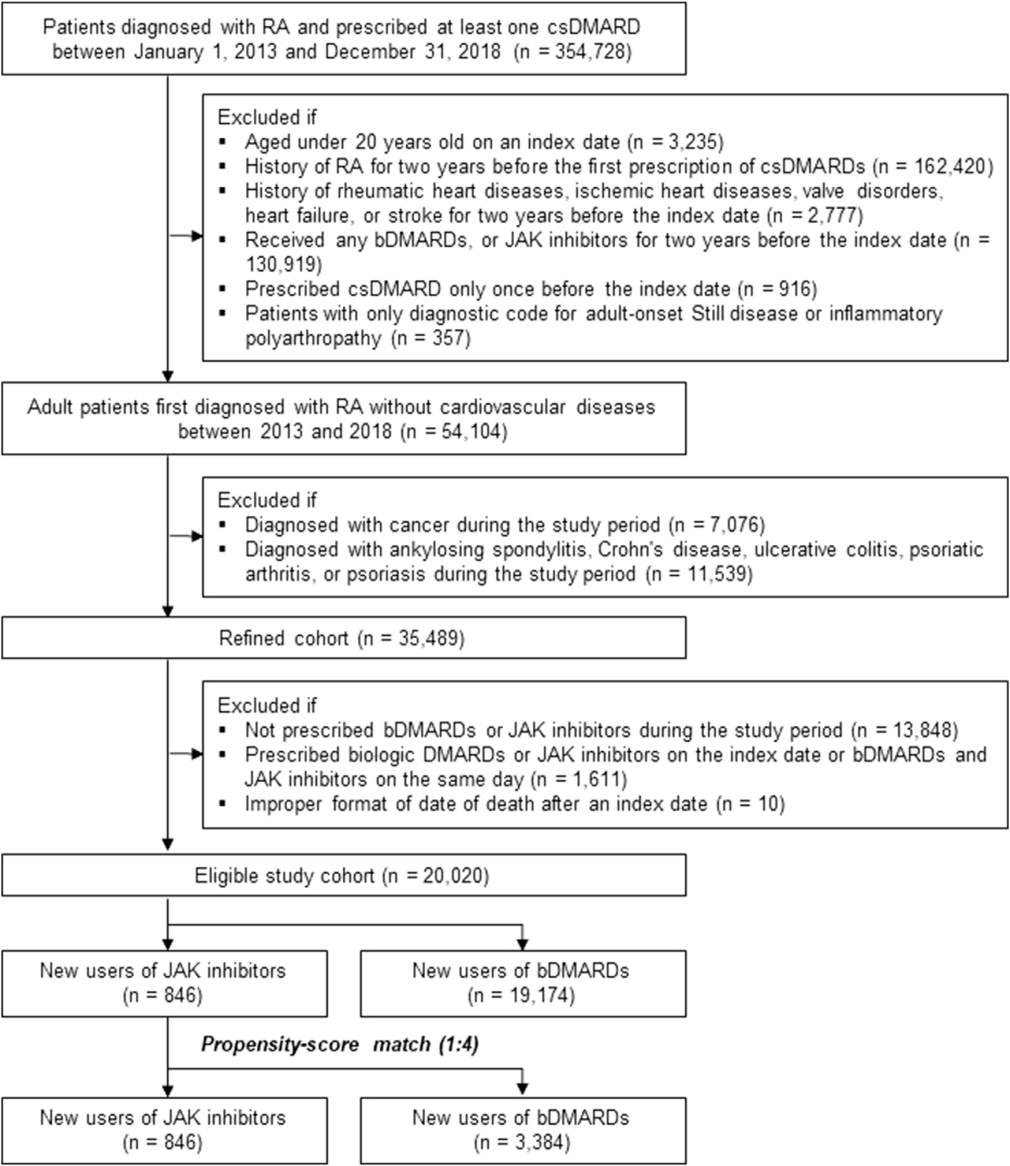
Study cohort selection process. Abbreviations: bDMARD, biologic disease-modifying antirheumatic drug; csDMARD, conventional synthetic disease-modifying antirheumatic drug; IHD, ischemic heart disease; JAK, Janus kinase; RA, rheumatoid arthritis.

Among the 354,728 patients with RA who were prescribed at least one csDMARD between 2013 and 2018, 334,708 patients were excluded according to the predefined exclusion criteria. The eligible study cohort included 20,020 patients newly diagnosed with RA, without underlying CVD, and with no recent prescription of bDMARDs and JAK inhibitors (846 JAK inhibitor users and 19,174 bDMARD users before PS matching, Figure 2).

As shown in Table 1, the proportion of methotrexate (81.3% vs. 78.0%) or tramadol (82.1% vs. 79.3%) users was higher in the bDMARD group than in the JAK inhibitor group during a year prior to the first prescription of bDMARDs or JAK inhibitors. Furthermore, more bDMARD users were compared to JAK inhibitor users with the increasing order of the index year. After 1:4 PS matching, 846 JAK inhibitor users were matched with 3,384 bDMARD users, and both groups were well balanced. The mean age of JAK inhibitor and bDMARD users was 48.1 ± 12.7 and 48.5 ± 12.6 years, respectively, and 91.2% patients were <65-year-old. Women accounted for approximately 71% of the study cohort. From 2014 to 2015, approximately 65% patients were first prescribed bDMARD or JAK inhibitors. The CCI score was ≤1 point in 94.6% patients, and more than 50% patients had a history of hypertension or dyslipidemia within a year of the first use of the study drugs. During the baseline assessment period, approximately 79% patients received methotrexate or hydroxychloroquine as csDMARDs, while NSAIDs or corticosteroids were prescribed to more than 90% patients. The average period from the first diagnosis of RA with a csDMARD to the commencement of a JAK inhibitor or a bDMARD was 6.5 months in both groups.

**TABLE 1:** Baseline characteristics before and after 1:4 propensity-score matching, number of patients (%).

Characteristics	Before propensity score matching			After propensity score matching (1:4)			
	JAK inhibitors (n = 846)	bDMARDs (n = 19,174)	*p*-value	JAK inhibitors (n = 846)	bDMARDs (n = 3,384)	*p*-value	SMD
Age, year, mean ± SD	48.1 ± 12.7	48.5 ± 12.6	0.377	48.1 ± 12.7	48.5 ± 12.6	0.352	0.033
<65 years	765 (90.4)	17,246 (89.9)	0.649	765 (90.4)	3,093 (91.4)	0.370	0.034
≥65 years	81 (9.6)	1,928 (10.1)		81 (9.6)	291 (8.6)		
Sex							
Male	249 (29.4)	5,159 (26.9)	0.105	249 (29.4)	991 (29.3)	0.933	0.003
Female	597 (70.6)	14,015 (73.1)		597 (70.6)	2,393 (70.7)		
Index year							
2014	279 (33.0)	8,216 (42.9)	<.001	279 (33.0)	1,103 (32.6)	0.963	0.008
2015	266 (31.4)	6,021 (31.4)		266 (31.4)	1,108 (32.7)		0.028
2016	159 (18.8)	3,119 (16.3)		159 (18.8)	598 (17.7)		0.029
2017	91 (10.8)	1,226 (6.4)		91 (10.8)	364 (10.8)		0
2018	51 (6.0)	592 (4.1)		51 (6.0)	211 (6.2)		0.009
Type of insurance							
Health insurance	828 (97.9)	18,773 (97.9)	0.943	828 (97.9)	3,311 (97.8)	0.958	0.002
Medical aid	18 (2.1)	401 (2.1)		18 (2.1)	73 (2.2)		
CCI score							
0	561(66.3)	12,262 (64.0)	0.153	561(66.3)	2,287 (67.6)	0.337	0.027
1	228 (27.0)	5,232 (27.3)		228 (27.0)	922 (27.3)		0.007
2	47 (5.6)	1,295 (6.8)		47 (5.6)	148 (4.4)		0.055
≥3	10 (1.2)	385 (2.0)		10 (1.2)	27 (0.8)		0.038
Comorbidities							
Hypertension	485 (57.3)	11,255 (58.7)	0.428	485 (57.3)	1,917 (56.7)	0.721	0.014
Dyslipidemia	437 (51.7)	9,981 (52.1)	0.820	437 (51.7)	1,715 (50.7)	0.612	0.019
Diabetes mellitus	189 (22.3)	4,595 (24.0)	0.278	189 (22.3)	718 (21.2)	0.477	0.027
Eye disorders	205 (24.2)	4,903 (25.6)	0.382	205 (24.2)	750 (22.2)	0.198	0.049
Osteoporosis	151 (17.9)	3,737 (19.5)	0.238	151 (17.9)	540 (16.0)	0.183	0.050
Anemia	47 (5.6)	1,107 (5.8)	0.790	47 (5.6)	167 (4.9)	0.461	0.028
Medications for RA							
csDMARDs							
Methotrexate	660 (78.0)	15,592 (81.3)	0.016	660 (78.0)	2,685 (79.3)	0.395	0.032
Hydroxy-chloroquine	668 (79.0)	15,227 (79.4)	0.749	668 (79.0)	2,665 (78.8)	0.895	0.005
Sulfasalazine	389 (46.0)	8,787 (45.8)	0.930	389 (46.0)	1,510 (44.6)	0.477	0.027
Leflunomide	328 (38.8)	8,065 (42.1)	0.058	328 (38.8)	1,311 (38.7)	0.987	0.001
Corticoste-roids	786 (92.9)	18,101 (94.4)	0.065	786 (92.9)	3,159 (93.4)	0.646	0.017
Cumulative dose,^a^ mean ± SD	33.2 ± 63.3	28.3 ± 74.1	0.070	31.5 ± 75.5	28.3 ± 74.1	0.311	0.043
NSAIDs	819 (96.8)	18,757 (97.8)	0.050	819 (96.8)	3,298 (97.5)	0.294	0.039
Tramadol	671 (79.3)	15,735 (82.1)	0.042	671 (79.3)	2,760 (81.6)	0.136	0.057
Other comedications							
Statins	249 (29.4)	5,690 (29.7)	0.880	249 (29.4)	1,012 (29.9)	0.788	0.011
Antidiabetics	189 (22.3)	4,595 (24.0)	0.278	189 (22.3)	718 (21.2)	0.477	0.027
Antihyper-tensives	401 (47.4)	9,398 (49.0)	0.358	401 (47.4)	1,613 (47.7)	0.890	0.005

Abbreviations: bDMARD, biologic disease-modifying antirheumatic drug; CCI, charlson comorbidity index; csDMARD, conventional synthetic disease-modifying antirheumatic drug; JAK, janus kinase; NSAID, non-steroidal anti-inflammatory drug; RA, rheumatoid arthritis; SD, standard deviation; SMD, standardized mean difference.

^a^ Prednisolone equivalent dose in milligrams.

### 3.2 MACEs associated with the use of JAK inhibitors

As shown in Table 2, the overall IR (95% CI) per 100 patient-years (PY) for composite MACEs after PS matching was 0.83 (0.31–1.81; 6/846 events) and 0.74 (0.53–1.02; 38/3,384 events) in the JAK inhibitor and bDMARD groups, respectively. The median time to onset of the first MACE was 9.14 and 60.71 weeks in the JAK inhibitor and bDMARD groups, respectively. Compared to the risk of MACEs in the bDMARD group, that in the JAK inhibitor group was 28% higher, but the difference was not statistically significant (adjusted HR: 1.28, 95% CI: 0.53–3.11).

**TABLE 2:** Risks of cardiovascular events in patients with RA treated with JAK inhibitors versus biologic DMARDs for the propensity score matched cohort.

	No. of patients	No. of events (%)	Time to onset (weeks), median (range)	PY	IR per 100 PY (95% CI)	Cardiovascular events	
						Unadjusted HR (95% CI)	Adjusted HR (95% CI)
Primary outcome							
MACE							
JAK inhibitors	846	6 (0.71)	9.14 (4.71–45.43)	720	0.83 (0.31–1.81)	1.27 (0.52–3.08)	1.28 (0.53–3.11)
Biologic DMARDs	3,384	38 (1.12)	60.71 (4.29–221.14)	5,126	0.74 (0.53–1.02)	References	
Secondary outcomes							
Myocardial infarction							
JAK inhibitors	846	0	NA	724	NA	NA	NA
Biologic DMARDs	3,384	8 (0.24)	57.93 (17.43–173.71)	5,148	0.16 (0.07–0.31)	References	
Ischemic stroke							
JAK inhibitors	846	0	NA	724	NA	NA	NA
Biologic DMARDs	3,384	0	NA	5,156	NA	References	
Coronary revascularization							
JAK inhibitors	846	2 (0.24)	13.64 (4.71–22.57)	722	0.28 (0.03–1.00)	0.96 (0.22–4.28)	0.95 (0.21–4.21)
Biologic DMARDs	3,384	18 (0.53)	94.71 (20.14–217.29)	5,142	0.35 (0.21–0.55)	References	
All-cause death							
JAK inhibitors	846	4 (0.47)	9.14 (6.43–45.43)	721	0.56 (0.15–1.42)	2.42 (0.73–7.99)	2.38 (0.72–7.90)
Biologic DMARDs	3,384	12 (0.53)	61.29 (4.29–196.00)	5,149	0.23 (0.12–0.41)	References	

Abbreviations: CI, confidence interval; DMARD, disease-modifying antirheumatic drug; IR, incidence rate; JAK, janus kinase; MACE, major adverse cardiovascular event; NA, not applicable; PY, patient-years; RA, rheumatoid arthritis.

None of the patient in the JAK inhibitor group experienced MI or ischemic stroke, while eight patients in the bDMARD group experienced MI only. The IRs (95% CI) of coronary revascularization were 0.28 (0.03–1.00) and 0.35 (0.21–0.55) per 100 PY, in the JAK inhibitor group and bDMARD groups, respectively. The IR (95% CI) of all-cause death was 2.4-fold higher in patients prescribed JAK inhibitors [0.56 (0.15–1.42)] than in those prescribed bDMARDs [0.23 (0.12–0.41)]. Regarding each component of MACEs, JAK inhibitors did not increase the HR of coronary revascularization and all-cause death compared to bDMARDs (adjusted HR [95% CI]: 0.95 [0.21–4.21] and 2.38 [0.72–7.90], respectively). The proportional hazard assumption was appropriate, as the *p*-value was greater than 0.05 in the goodness-of-fit test.

**FIGURE 3:**
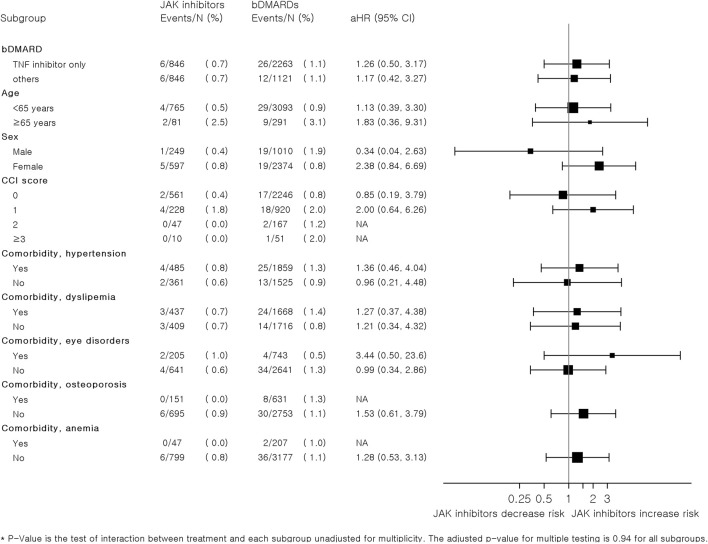
Subgroup analysis of hazard ratios for major adverse cardiovascular events associated with JAK inhibitors and bDMARDs in a 1:4 variable ratio propensity score-matched cohort of patients with RA. Abbreviations: bDMARD, biologic disease-modifying antirheumatic drug; csDMARD, conventional synthetic disease-modifying antirheumatic drug; IHD, ischemic heart disease; JAK, Janus kinase; RA, rheumatoid arthritis.

Figure 3 summarizes the results of subgroup analyses according to the types of bDMARDs, age, sex, CCI score, and comorbidities. Overall, there were no significant differences in the risk of MACEs between JAK inhibitors and bDMARDs in each subgroup. However, the risk of CVD associated with JAK inhibitors tended to increase compared to that with bDMARDs in patients aged ≥65 years (adjusted HR: 1.83, 95% CI: 0.36–9.31). Women who were prescribed JAK inhibitors had higher risks than those who were prescribed bDMARDs (adjusted HR: 2.38, 95% CI: 0.84–6.69); however, the adjusted HR (95% CI) for men was only 0.34 (0.04–2.63). The HRs were higher in patients with comorbidities, such as hypertension, or eye disorder, in comparison to those without the comorbidities.

As shown in Table 3, the risk of MACEs with JAK inhibitors compared to that with bDMARDs did not significantly differ according to the different permissible treatment gaps. When we defined the treatment gap as 180 and 90 days, the adjusted HRs (95% CI) were 1.59 (0.52–4.91) and 1.45 (0.54–3.94), respectively.

**TABLE 3:** Sensitivity analysis in the propensity-score matched cohort.

	No. of subjects	No. of events (%)	Time to onset (weeks), median (range)	PY	IR per 100 PY (95% CI)	MACEs	
						Unadjusted HR (95% CI)	Adjusted HR (95% CI)
Treatment gap, 180 days							
JAK inhibitors	846	5 (0.59)	7.00 (4.71–22.57)	545	0.92 (0.30–2.14)	1.61 (0.52–4.93)	1.59 (0.52–4.91)
Biologic DMARDs	3,384	22 (0.65)	31.14 (4.29–221.14)	3,811	0.58 (0.36–0.87)	References	
Treatment gap, 90 days							
JAK inhibitors	846	4 (0.47)	6.71 (4.71–11.29)	435	0.92 (0.25–2.35)	1.43 (0.53–3.85)	1.45 (0.54–3.94)
Biologic DMARDs	3,384	16 (0.47)	38.36 (4.29–173.71)	3,087	0.52 (0.30–0.84)	References	

Abbreviations: CI, confidence interval; DMARD, disease-modifying antirheumatic drug; IR, incidence rate; JAK, janus kinase; MACE, major adverse cardiovascular event; PY, patient-years.

## 4 Discussion

To the best of our knowledge, this is the first large population-based cohort study to evaluate the impact of JAK inhibitors on MACEs compared to that of bDMARDs in routine care patients with early diagnosed RA and no underlying CVD in Asia.

Overall, JAK inhibitors, compared to bDMARDs, were not associated with the risk of MACEs in this real-world setting (adjusted HR: 1.28, 95% CI: 0.53–3.11) in newly diagnosed patients with RA with an average disease duration of 6.5 months. Among Asian patients with an average RA duration of 3.2 years, the CV risk was not significantly increased compared to TNF inhibitors [i.e., risk ratio (95% CI), 1.12 (0.64–1.95); Tong et al., 2023]. In American patients with RA and no previous history of CVD, the risk of CVD was not significantly different between tofacitinib and TNF inhibitor users although a decreased risk was reported (pooled weighted HR: 0.81, 95% CI: 0.61–1.07) (Khosrow-Khavar et al., 2022). This might be due to the different definition of the composite CV outcomes, which included hospitalization for MI or stroke in the previous study (Kip et al., 2008); however, coronary revascularization and all-cause death were additionally considered in our study. We defined MACEs considering the most common components of MACEs used in RCTs and observational studies (Kip et al., 2008; Bosco et al., 2021). The results of subgroup analyses showed that the risks of composite CV events associated with JAK inhibitors tended to increase in patients aged ≥65 years and those with a CV-related comorbidities such as hypertension, although the difference was statistically non-significant. This is concordant with the findings of previous studies (Khosrow-Khavar et al., 2022; Ytterberg et al., 2022).

Several RCTs and observational studies have reported inconsistent results. The prospective ORAL surveillance trial revealed that MACEs occurred more often with tofacitinib than with a TNF inhibitor in aged patients with RA and underlying CV risk factors; thus, it might not capture the real-world risk for MACEs in patients without underlying CVDs at treatment initiation (Ytterberg et al., 2022). This was in contrast to the conclusion of previous studies in which JAK inhibitors did not significantly change the CV outcomes and their IRs were unchanged for up to 9.5 years (Xie W. et al., 2019; Cohen et al., 2020). Moreover, several observational studies found no increased CV risks with JAK inhibitors in patients with RA treated regardless of the presence of CV risk factors (Kremer et al., 2021; Khosrow-Khavar et al., 2022). Therefore, continuing research to better understand the CV risks of this important treatment option is recommended in a wide range of patients with RA.

The IRs of MACEs in Asian patients without underlying CVDs (0.83 and 0.74 per 100 PY with JAK inhibitors and bDMARDs, respectively) were similar to those reported in Western patients without underlying CVDs (0.87 and 0.79 per 100 PY with tofacitinib and TNF inhibitors, respectively) (Khosrow-Khavar et al., 2022). However, considering the differences in the definition of MACEs between the studies, as mentioned above, the incidence of MACEs associated with the use of JAK inhibitors or bDMARDs was relatively low in Korean patients with RA compared to that in Westerner patients; this is consistent with the findings of previous studies reporting a low risk of CVDs in Asians (Meadows et al., 2011; Post et al., 2022). The incidences of death from any cause was higher with JAK inhibitors than with bDMARDs in our study (IR of 0.56 and 0.23 with JAK inhibitor and bDMARD, respectively), similar to the findings reported by Khosrow-Khavar et al. (IR of 1.95 and 1.41 with tofacitinib and a TNF inhibitor, respectively) (Khosrow-Khavar et al., 2022). In the ORAL surveillance trial, the HR (95% CI) for all-cause death was significantly high with tofacitinib compared to that with a TNF inhibitor (2.37 [1.34–4.18]) (Ytterberg et al., 2022). It has been reported that bDMARDs may reduce the risk of MACEs, particularly mortality related to coronary heart diseases, in patients with RA (Myasoedova et al., 2017; Xie F. et al., 2019; Provan et al., 2020; Singh et al., 2020). This may be due to a positive impact of these modern treatment strategies on the RA severity and mortality. The causal relationship between the use of JAK inhibitors and CV risk, including death, is unknown. Considering the relatively high mortality rate from any cause in JAK inhibitors, close monitoring and further research into the causal relationship are required.

The time to onset in the bDMARD group was longer in Korean patients without CVDs (12.1 months after the use of bDMARDs) than in American patients (6.1 months); this was likely because the analysis of Western patients included patients with and without CVDs, and the East Asian population exhibited a relatively lower CV risk than the Western population (Meadows et al., 2011; Khosrow-Khavar et al., 2022; Post et al., 2022). While CVD risk has been associated with various factors such as age, sex and chronic diseases, RA diagnosis itself has also been linked to an increased likelihood of developing CVDs. Previous study indicated that the risk of CVD increased shortly after the diagnosis of RA, mostly within a year of the clinical onset of RA (Kerola et al., 2012). In consideration of the recommended initiation time of the bDMARDs or JAK inhibitors after the diagnosis of RA (i.e., at least 6 months), the timeframe for CVD onset associated with these medications may align with the natural history of CVDs in RA patients (Smolen et al., 2020) In JAK inhibitor users, the median time to onset (range) of MACEs was short (1.8 [0.9–9.1] months). In contrast, it was reported that the median time to CV events after tofacitinib use was 5.1 months in Western patients (Khosrow-Khavar et al., 2022). Studies on ethnic differences in the time to drug-induced CV events are limited. Since it is the first study to demonstrate the relatively reduced onset time to the event in Asian users of JAK inhibitors, it is necessary to monitor continuously and expand the related research in Asian patients.

As there was no significant difference among JAK inhibitors regarding the occurrence of CV or thromboembolic events, we analyzed all JAK inhibitors approved for the treatment of RA in Korea until 2018 (i.e., tofacitinib and baricitinib) (Alves et al., 2021). Additionally, we used the bDMARD group as a control group because a bDMARD or a JAK inhibitor was recommended as a second-line agent for patients with poor RA prognostic factors who failed with the first treatment with csDMARD based on the EULAR guideline (Smolen et al., 2020). There was no significant difference in the risk of MACEs associated with the use of TNF and non-TNF inhibitors in patients with RA (Singh et al., 2020). In the subgroup analysis of this study, the HR of JAK inhibitors compared with patients received only TNF inhibitors was similar to that of patients prescribed non-TNF inhibitors.

Although our results highlight a potentially insightful relationship between the use of JAK inhibitors and CV risks in the real world using large-scale administrative data, our study has several limitations. First, there were no clinical laboratory results to evaluate the disease severity of RA at the index date, which might affect the CV risk (Crowson et al., 2013; Health Insurance Review and Assessment Service, 2021a). Therefore, we included patients who were first administered a JAK inhibitor or bDMARD after being newly diagnosed with RA to balance the RA severity and duration. The period from the first diagnosis of RA to the first prescription of the study drug was similar in both groups. It has been reported that the development of CVD in Asian patients with RA might be influenced more by high-grade systemic inflammation compared to individual CVD risk factors, which tend to have a greater impact in non-Asian populations (You et al., 2011) Therefore, further studies are needed to evaluate the risk of JAK inhibitors in RA patients with advanced disease. Second, coronary artery calcium scores, a known predictive factor for coronary heart disease, could not be assessed due to the nature of the administrative data (Polonsky et al., 2010). Further research is needed using clinical data, including electronic medical records. Third, we used all-cause death, not CV-related death, as a component of MACEs. The balance between the use of all-cause mortality and cardiac-only mortality was approximately equal (Kip et al., 2008; Bosco et al., 2021). However, as it has not been validated to confirm CVD-related death using the ICD-10 code of the claim data from the HIRA, we used all-cause death instead of CVD-related death for the definition of MACEs (Bosco et al., 2021). Lastly, the interpretation of this study results had some caution for RA patients who had longer duration because enrolled patients in 2014 (earliest index date) had maximum follow-up period of 5 years.

Taken together, this large population-based study revealed that, compared to the use of bDMARDs, the use of JAK inhibitors was not significantly associated with the occurrence of MACEs in Asian patients with RA and no underlying CVDs. The results remained robust across various sensitive analyses.

## Data Availability

The original contributions presented in the study are included in the article/supplementary material, further inquiries can be directed to the corresponding author.

## References

[B1] Alves C. Penedones A. Mendes D. Marques F. B. (2022). Risk of cardiovascular and venous thromboembolic events associated with Janus kinase inhibitors in rheumatoid arthritis: a systematic review and network meta-analysis. J. Clin. Rheumatol. 28 (2), 69–76. 10.1097/RHU.0000000000001804 34741000

[B2] Avina-Zubieta J. A. Thomas J. Sadatsafavi M. Lehman A. J. Lacaille D. (2012). Risk of incident cardiovascular events in patients with rheumatoid arthritis: a meta-analysis of observational studies. Ann. Rheum. Dis. 71 (9), 1524–1529. 10.1136/annrheumdis-2011-200726 22425941

[B3] Baldini C. Moriconi F. R. Galimberti S. Libby P. De Caterina R. (2021). The JAK-STAT pathway: an emerging target for cardiovascular disease in rheumatoid arthritis and myeloproliferative neoplasms. Eur. Heart. J. 42 (42), 4389–4400. 10.1093/eurheartj/ehab447 34343257 PMC8572559

[B4] Bosco E. Hsueh L. McConeghy K. W. Gravenstein S. Saade E. (2021). Major adverse cardiovascular event definitions used in observational analysis of administrative databases: a systematic review. BMC Med. Res. Methodol. 21 (1), 241. 10.1186/s12874-021-01440-5 34742250 PMC8571870

[B5] Cohen S. B. Tanaka Y. Mariette X. Curtis J. R. Lee E. B. Nash P. (2020). Long-term safety of tofacitinib up to 9.5 years: a comprehensive integrated analysis of the rheumatoid arthritis clinical development programme. RMD Open 6 (3), e001395. 10.1136/rmdopen-2020-001395 33127856 PMC7722371

[B6] Crowson C. S. Liao K. P. Davis J. M. 3rd Solomon D. H. Matteson E. L. Knutson K. L. (2013). Rheumatoid arthritis and cardiovascular disease. Am. Heart. J. 166 (4), 622–628. 10.1016/j.ahj.2013.07.010 24093840 PMC3890244

[B7] European Medicines Agency (2022). Xeljanz (tofacitinib), summary of product characteristics . https://www.ema.europa.eu/en/medicines/human/EPAR/xeljanz#product-information-section (Accessed July 16, 2022).

[B8] Gadina M. Le M. T. Schwartz D. M. Silvennoinen O. Nakayamada S. Yamaoka K. (2019). Janus kinases to jakinibs: from basic insights to clinical practice. Rheumatol. Oxf. 58 (1), i4–i16. 10.1093/rheumatology/key432 PMC6657570 30806710

[B9] Health Insurance Review and Assessment Service (2019). Healthcare insurance coverage criteria and methods – pharmaceuticals. https://www.hira.or.kr/eng/main.do.

[B10] Health Insurance Review and Assessment Service (2021a). Function and role of health insurance review and assessment Service. https://www.hira.or.kr/eng/main.do.

[B11] Health Insurance Review and Assessment Service (2021b). Healthcare big data. https://www.hira.or.kr/eng/main.do.

[B12] Health Insurance Review and Assessment Service (2022). Healthcare bigdata hub . http://opendata.hira.or.kr/op/opc/olap3thDsInfo.do (Accessed August 20, 2022).

[B13] Kerola A. M. Kauppi M. J. Kerola T. Nieminen T. V. (2012). How early in the course of rheumatoid arthritis does the excess cardiovascular risk appear? Ann. Rheum. Dis. 71 (10), 1606–1615. 10.1136/annrheumdis-2012-201334 22736093

[B14] Khosrow-Khavar F. Kim S. C. Lee H. Lee S. B. Desai R. J. (2022). Tofacitinib and risk of cardiovascular outcomes: results from the Safety of TofAcitinib in Routine care patients with Rheumatoid Arthritis (STAR-RA) study. Ann. Rheum. Dis. 81 (6), 798–804. 10.1136/annrheumdis-2021-221915 35027405 PMC9117457

[B15] Kim S. Y. Servi A. Polinski J. M. Mogun H. Weinblatt M. E. Katz J. N. (2011). Validation of rheumatoid arthritis diagnoses in health care utilization data. Arthritis. Res. Ther. 13 (1), R32. 10.1186/ar3260 21345216 PMC3241376

[B16] Kip K. E. Hollabaugh K. Marroquin O. C. Williams D. O. (2008). The problem with composite end points in cardiovascular studies: the story of major adverse cardiac events and percutaneous coronary intervention. J. Am. Coll. Cardiol. 51 (7), 701–707. 10.1016/j.jacc.2007.10.034 18279733

[B17] Korean Ministry of Food and Drug Safety (2022). Drug information system . https://nedrug.mfds.go.kr/(Accessed July 16, 2022).

[B18] Kremer J. M. Bingham C. O. 3rd Cappelli L. C. Greenberg J. D. Madsen A. M. Geier J. (2021). Postapproval comparative safety study of tofacitinib and biological disease-modifying antirheumatic drugs: 5-year results from a United States-based rheumatoid arthritis registry. ACR Open. Rheumatol. 3 (3), 173–184. 10.1002/acr2.11232 33570260 PMC7966883

[B19] Meadows T. A. Bhatt D. L. Cannon C. P. Gersh B. J. Rother J. Goto S. (2011). Ethnic differences in cardiovascular risks and mortality in atherothrombotic disease: insights from the Reduction of Atherothrombosis for Continued Health (REACH) registry. Mayo Clin. Proc. 86 (10), 960–967. 10.4065/mcp.2011.0010 21964173 PMC3184025

[B20] Myasoedova E. Gabriel S. E. Matteson E. L. Davis J. M. 3rd Therneau T. M. Crowson C. S. (2017). Decreased cardiovascular mortality in patients with incident rheumatoid arthritis (RA) in recent years: dawn of a new era in cardiovascular disease in RA? J. Rheumatol. 44 (6), 732–739. 10.3899/jrheum.161154 28365576 PMC5457313

[B21] Park J. Kwon S. Choi E.-K. Choi Y.-j. Lee E. Choe W. (2019). Validation of diagnostic codes of major clinical outcomes in a National Health Insurance database. Int. J. Arrhythm. 20 (1), 5. 10.1186/s42444-019-0005-0

[B22] Parsons L. S. (2001). Reducing bias in a propensity score matched-pair sample using Greedy matching techniques. https://support.sas.com/resources/papers/proceedings/proceedings/sugi26/p214-26.pdf.

[B23] Polonsky T. S. McClelland R. L. Jorgensen N. W. Bild D. E. Burke G. L. Guerci A. D. (2010). Coronary artery calcium score and risk classification for coronary heart disease prediction. JAMA 303 (16), 1610–1616. 10.1001/jama.2010.461 20424251 PMC3033741

[B24] Post W. S. Watson K. E. Hansen S. Folsom A. R. Szklo M. Shea S. (2022). Racial and ethnic differences in all-cause and cardiovascular disease mortality: the MESA study. Circulation 146 (3), 229–239. 10.1161/CIRCULATIONAHA.122.059174 35861763 PMC9937428

[B25] Provan S. A. Lillegraven S. Sexton J. Angel K. Austad C. Haavardsholm E. A. (2020). Trends in all-cause and cardiovascular mortality in patients with incident rheumatoid arthritis: a 20-year follow-up matched case-cohort study. Rheumatol. Oxf. 59 (3), 505–512. 10.1093/rheumatology/kez371 31504942

[B26] Singh J. A. (2022). Risks and benefits of Janus kinase inhibitors in rheumatoid arthritis - past, present, and future. N. Engl. J. Med. 386 (4), 387–389. 10.1056/NEJMe2117663 35081285

[B27] Singh S. Fumery M. Singh A. G. Singh N. Prokop L. J. Dulai P. S. (2020). Comparative risk of cardiovascular events with biologic and synthetic disease-modifying antirheumatic drugs in patients with rheumatoid arthritis: a systematic review and meta-analysis. Arthritis. Care. Res. Hob. 72 (4), 561–576. 10.1002/acr.23875 PMC6745288 30875456

[B28] Smolen J. S. Aletaha D. Barton A. Burmester G. R. Emery P. Firestein G. S. (2018). Rheumatoid arthritis. Nat. Rev. Dis. Prim. 4 (1), 18001. 10.1038/nrdp.2018.1 29417936

[B29] Smolen J. S. Landewé R. B. M. Bijlsma J. W. J. Burmester G. R. Dougados M. Kerschbaumer A. (2020). EULAR recommendations for the management of rheumatoid arthritis with synthetic and biological disease-modifying antirheumatic drugs: 2019 update. Ann. Rheum. Dis. 79 (6), 685–699. 10.1136/annrheumdis-2019-216655 31969328

[B30] Song Y.-K. Song J. Kim K. Kwon J.-W. (2022). Potential adverse events reported with the Janus kinase inhibitors approved for the treatment of rheumatoid arthritis using spontaneous reports and online patient reviews. Front. Pharmacol. 12, 792877. 10.3389/fphar.2021.792877 35087406 PMC8787189

[B31] Takabayashi K. Ando F. Ikeda K. Fujita S. Nakajima H. Hanaoka H. (2021). Trend in prescription and treatment retention of molecular-targeted drugs in 121,131 Japanese patients with rheumatoid arthritis: a population-based real-world study. Mod. Rheumatol. 32 (5), 857–865. 10.1093/mr/roab126 34907436

[B32] Taylor P. C. Takeuchi T. Burmester G. R. Durez P. Smolen J. S. Deberdt W. (2022). Safety of baricitinib for the treatment of rheumatoid arthritis over a median of 4.6 and up to 9.3 years of treatment: final results from long-term extension study and integrated database. Ann. Rheum. Dis. 81 (3), 335–343. 10.1136/annrheumdis-2021-221276 34706874 PMC8862028

[B33] Tong X. Shen C. Y. Jeon H. L. Li Y. Shin J. Y. Chan S. C. (2023). Cardiovascular risk in rheumatoid arthritis patients treated with targeted synthetic and biological disease-modifying antirheumatic drugs: a multi-centre cohort study. J. Intern. Med. 294 (3), 314–325. 10.1111/joim.13681 37282790

[B34] Tsao C. W. Aday A. W. Almarzooq Z. I. Alonso A. Beaton A. Z. Bittencourt M. S. (2022). Heart disease and stroke statistics-2022 update: a report from the American Heart Association. Circulation 145 (8), e153–e639. 10.1161/CIR.0000000000001052 35078371

[B35] Us Food and Drug Administration (2013). Guidance for industry and FDA staff: best practices for conducting and reporting pharmacoepidemiologic safety studies using electronic healthcare data sets. https://www.fda.gov/regulatory-information/search-fda-guidance-documents/best-practices-conducting-and-reporting-pharmacoepidemiologic-safety-studies-using-electronic.

[B36] Us Food and Drug Administration (2021b). Xeljanz (tofacitinib citrate) label . https://www.accessdata.fda.gov/scripts/cder/daf/index.cfm?event=overview.process&ApplNo=203214 (Accessed July 16, 2022).

[B37] Us Food and Drug Administration (2021a). Drugs@FDA: FDA-approved drugs . https://www.accessdata.fda.gov/scripts/cder/daf/index.cfm?event=BasicSearch.process (Accessed September 16, 2021).

[B38] Won S. Cho S. K. Kim D. Han M. Lee J. Jang E. J. (2018). Update on the prevalence and incidence of rheumatoid arthritis in Korea and an analysis of medical care and drug utilization. Rheumatol. Int. 38 (4), 649–656. 10.1007/s00296-017-3925-9 29302803

[B39] World Health Organization (2019). ICD-10 version:2019 . https://icd.who.int/browse10/2019/en (Accessed January 5, 2022).

[B40] Xie F. Yun H. Levitan E. B. Muntner P. Curtis J. R. (2019a). Tocilizumab and the risk of cardiovascular disease: direct comparison among biologic disease-modifying antirheumatic drugs for rheumatoid arthritis patients. Arthritis. Care. Res. Hob. 71 (8), 1004–1018. 10.1002/acr.23737 30175897

[B41] Xie W. Huang Y. Xiao S. Sun X. Fan Y. Zhang Z. (2019b). Impact of Janus kinase inhibitors on risk of cardiovascular events in patients with rheumatoid arthritis: systematic review and meta-analysis of randomised controlled trials. Ann. Rheum. Dis. 78 (8), 1048–1054. 10.1136/annrheumdis-2018-214846 31088790

[B42] Yeom H. Kang D. R. Cho S. K. Lee S. W. Shin D. H. Kim H. C. (2015). Admission route and use of invasive procedures during hospitalization for acute myocardial infarction: analysis of 2007-2011 National Health Insurance database. Epidemiol. Health. 37, e2015022. 10.4178/epih/e2015022 25968116 PMC4459111

[B43] Ytterberg S. R. Bhatt D. L. Mikuls T. R. Koch G. G. Fleischmann R. Rivas J. L. (2022). Cardiovascular and cancer risk with tofacitinib in rheumatoid arthritis. N. Engl. J. Med. 386 (4), 316–326. 10.1056/NEJMoa2109927 35081280

